# Climate Change Could Increase the Geographic Extent of Hendra Virus Spillover Risk

**DOI:** 10.1007/s10393-018-1322-9

**Published:** 2018-03-19

**Authors:** Gerardo Martin, Carlos Yanez-Arenas, Carla Chen, Raina K. Plowright, Rebecca J. Webb, Lee F. Skerratt

**Affiliations:** 1 One Health Research Group, College of Public Health, Medical and Veterinary Sciences, James Cook University, Townsville, QLD Australia; 2 Laboratorio de Conservación de la Biodiversidad, Parque Científico y Tecnológico de Yucatán, Universidad, Universidad Nacional Autónoma de México, Mérida, Yucatán Mexico; 3 Australian Institute of Marine Sciences, Townsville, QLD Australia; 4 Bozeman Disease Ecology Lab, Department of Microbiology and Immunology, Montana State University, Bozeman, MT USA; 5 Guadalupe Victoria, Mexico; 6 Ecological Health Research Group, Department of Infectious Disease Epidemiology, Imperial College London, St. Mary’s campus, Praed Street, London, W2 1NY UK

**Keywords:** spillover, climate change, risk, Hendra virus, flying foxes, horses

## Abstract

Disease risk mapping is important for predicting and mitigating impacts of bat-borne viruses, including Hendra virus (Paramyxoviridae:*Henipavirus*), that can spillover to domestic animals and thence to humans. We produced two models to estimate areas at potential risk of HeV spillover explained by the climatic suitability for its flying fox reservoir hosts, *Pteropus alecto* and *P. conspicillatus*. We included additional climatic variables that might affect spillover risk through other biological processes (such as bat or horse behaviour, plant phenology and bat foraging habitat). Models were fit with a Poisson point process model and a log-Gaussian Cox process. In response to climate change, risk expanded southwards due to an expansion of *P. alecto* suitable habitat, which increased the number of horses at risk by 175–260% (110,000–165,000). In the northern limits of the current distribution, spillover risk was highly uncertain because of model extrapolation to novel climatic conditions. The extent of areas at risk of spillover from *P. conspicillatus* was predicted shrink. Due to a likely expansion of *P. alecto* into these areas, it could replace *P. conspicillatus* as the main HeV reservoir. We recommend: (1) HeV monitoring in bats, (2) enhancing HeV prevention in horses in areas predicted to be at risk, (3) investigate and develop mitigation strategies for areas that could experience reservoir host replacements.

**Electronic supplementary material:**

The online version of this article (10.1007/s10393-018-1322-9) contains supplementary material, which is available to authorized users.

## Introduction

Emerging zoonotic diseases account for close to 13% of known human pathogens (Taylor et al. 2001; Woolhouse and Gowtage-Sequeria 2005). These diseases along with other emerging pathogens that affect crops and domestic animals can have extensive socio-economic consequences (Jones et al. 2008), especially when they adapt to and transmit among their new hosts (Taylor et al. 2001). Four diseases that have spilled over from bats to humans and have resulted in epidemics are Ebola and Marburg viruses in Africa (Leroy et al. 2005) and severe acute respiratory syndrome (SARS) *Coronavirus* and Nipah virus in east Asia (Chua 2003; He et al. 2004; Leroy et al. 2005; Li et al. 2005; Wang and Eaton 2007). Some of these outbreaks have had long-term devastating consequences, from the loss of thousands of human lives to the collapse of the already imperilled public health systems that prevent, control and treat other diseases (Chang et al. 2004; Plucisnki et al. 2015).

Hendra virus (HeV, Paramyxoviridae:*Henipavirus*) is another bat-borne virus that spills over into domestic animals, in its case horses, and then people with high case fatality rates of 50–75% (Halpin et al. 2011; Smith et al. 2014; Edson et al. 2015; Martin et al. 2016). It was discovered in 1994 in a Brisbane suburb in Queensland, Australia, with two of the four Australian mainland flying fox species, *Pteropus alecto* and *P. conspicillatus*, as its major reservoir hosts (Halpin et al. 2011; Smith et al. 2014; Edson et al. 2015; Martin et al. 2016), although antibodies against HeV are commonly found in *P. scapulatus* and *P. poliocephalus* (Young et al. 1996; Plowright et al. 2008). HeV is closely related to Nipah virus, which is also a *Henipavirus* from pteropodid bats. Nipah virus occurs in east Asia and spills over to pigs and humans, where it has been able to cause epidemic disease outbreaks with case fatality rates close to 41% in humans (Chong et al. 2002; Chua 2003). In Bangladesh, spillover occurs directly to humans with even higher case fatality rates (Luby et al. 2009). The proven ability of henipaviruses to cause epidemic outbreaks with high case fatality rates make spillover mitigation highly necessary.

Mitigation and prevention of impacts of disease spillover depends on our understanding of the transmission process and ability to predict it (Plowright et al. 2017). Mechanistic models of infectious diseases have proven useful frameworks to make informed decisions towards controlling and mitigating the impacts of epidemics (Wickwire 1977). These methods require high-quality longitudinal data rarely available for pathogens that originate in wild animals (Woodroffe 1999).The poor understanding of HeV dynamics in bats (Plowright et al. 2015) limit our ability to directly predict HeV levels in those populations. Nevertheless, prediction can be made with alternative methods to mechanistic models at lower spatial and temporal resolutions. These methods are based on readily available data and can be used to model the response of the system of interest (Peterson 2006).

One approach to identify areas at risk from emerging infectious diseases is to model the ecological niche of the causal agent and its reservoir host with spatiality explicit climatic data, and to use the model to predict their geographic distribution (Escobar and Craft 2016). The process of niche modelling consists of relating the climatic conditions of locations where organisms are able to breed and persist with the prevailing climatic conditions of areas where species could occur (Soberón et al. 2005). The relationships between a species’ presence and climate are usually established with statistical models that ultimately represent a measure of environmental suitability. The spatial representation of environmental suitability helps visualisation of the model’s estimates in the form of maps (Peterson 2006). Assuming that the organisms’ niches being modelled do not undergo a climatic niche shift, models can be used to predict future distributions under climate change scenarios (Pearman et al. 2008). For instance, using these methods many diseases have been predicted to impact wider areas with climate change, expanding or shifting from tropical to subtropical areas (Lafferty 2009). Therefore, identifying areas at risk and anticipating the potential impacts of climate change on HeV spillover is critical to adequately allocate resources and mitigate risk.

Ecological niche modelling has been applied with varying degrees of success to investigate the distribution of the zoonotic niches of bat-borne viruses. For instance when Peterson et al. (2004) initially predicted areas at risk of Marburg disease spillover in Africa, left out wide areas that were later on shown to be at risk in updated models with improved methods and data (Peterson et al. 2006; Pigott et al. 2015). Previous ecological niche modelling studies of *Henipavirus* spillover systems have focused on answering ecological and epidemiological questions (Hahn et al. 2014), identifying reservoir hosts (Martin et al. 2016); identifying new populations at risk (Walsh 2015); or generating broad predictions of risk (Daszak et al. 2013). While their contribution towards improving our understanding has been valuable, none have focused on forecasting areas at risk of spillover in time, which is essential to anticipate the effects of climate change and inform public health measures (Braks et al. 2013).

In order to be able to predict the consequences of climate change, models must rely on climatic data that can be projected into the future, such as those resulting from global circulation models (Hijmans et al. 2005; Beaumont et al. 2008; Wiens et al. 2009). Empirical evidence suggests that HeV spillover is related to climate by several different mechanisms acting at different temporal and spatial scales. From broad to fine: the spatial and temporal abundance patterns of HeV reservoir hosts, flying foxes, are related to climatic suitability (Martin et al. 2016); the spatial dynamics of bats are largely governed by food resources that are dependent on climate (Hudson et al. 2010; Giles et al. 2016); the levels of HeV shedding may be linked to low food productivity and availability after severe weather events (Plowright et al. 2008; McFarlane et al. 2011; Páez et al. 2017; Peel et al. 2017); and lastly HeV survival in microclimates which might facilitate indirect transmission, is also dependent on climate (Martin et al. 2015, 2017).

In this study we present two models that estimate the areas at risk of Hendra virus spillover to horses under current and future climatic conditions. The models represent the climatic requirements for the presence of HeV’s reservoir hosts and the climatic conditions that have facilitated HeV’s transmission to horses. We used current and predicted future climatic conditions to project the statistical models and predict areas that could be at risk now and by year 2050 according to two representative greenhouse gas concentration pathways.

## Methods

Ecological niche models often use presence only data, resulting in the extensive use of algorithmic modelling (Elith et al. 2011). A well-known disadvantage of these methods is their potential for complexity. Recent efforts have made the application of better understood techniques like generalised linear models possible for presence only data, in the form of Poisson point process models (Renner and Warton 2013; Renner et al. 2015). Taking advantage of these statistically transparent frameworks, we modelled the risk of HeV spillover as a Poisson point process, including a log-Gaussian Cox process to model spatial autocorrelation in a Bayesian hierarchical inference framework. This method allowed us to use the entire HeV spillover database (55 events between 1994 and 2015) without thinning to control spatial autocorrelation (Diggle et al. 2013; Boria et al. 2014). The models represent the relationship between the number of spillover events per unit area, the climatic suitability for reservoir host species, and the climatic niche (temperature and precipitation) over which spillover events have occurred to date.

**Figure 1:**
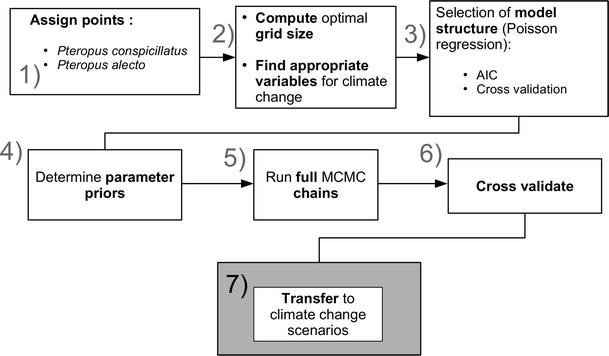
Workflow to construct the models.

We took the following steps to build these models: (1) assigned presence points to the most likely reservoir host species present at spillover locations, (2) computed the optimal size of spatial units and determined appropriate explanatory climatic variables, (3) selected the model structure (linear and quadratic terms and interactions with AIC and cross-validation), (4) selected priors for the Bayesian model, (5) fitted the Bayesian model, (6) cross-validated, and (7) transferred models to climate change scenarios (Fig. 1).

### Assigning Spillover Events to Reservoir Host Species

HeV spills over to horses from two of the four Australian flying fox species (Smith et al. 2014; Edson et al. 2015; Martin et al. 2016). We treated these species as two separate reservoir host systems (Smith et al. 2014; Edson et al. 2015; Martin et al. 2018) that were geographically limited to the areas colonisable by *P. alecto* and *P. conspicillatus* (Soberón et al. 2005; Martin et al. 2016). The colonisable areas comprise the climatic regions of Australia (obtained from the bureau of meteorology, http:www.bom.gov.au) that contain at least one presence record of each reservoir host.

To assign spillover events to either system (*P. alecto*, north and south, and *P. conspicillatus*, strictly north), we extracted the Maxent model’s relative probability of presence of the two flying fox species at the location of spillover (Martin et al. 2016). The location points were assigned to the relevant reservoir host system based on a higher relative probability of presence value for that species. To this data, from the remaining location points that did not have greater occurrence probability for either species, we added the points with the top 5% of occurrence probability (two spillover events) for that species at spillover locations. With this final step, we allowed for ambiguity of the most likely reservoir species that acted as the source of infection in horses.

### Estimating Spatial Resolution and Selecting Explanatory Climatic Variables

Point process models relate the number of points (intensity) of presence locations to spatial units. Before a model was fitted, we needed to determine the optimal size of the spatial units in which spillover intensity would be regressed against a set of explanatory variables. To estimate the optimal computational resolution, we used a minimum contrast method. Briefly, this method consists of comparing the bandwidth of a nonparametric field and the theoretical model that describes the spatial process of the data (Davies and Hazelton 2013). The spatial resolution that minimises the error between the methods is optimal.

The expected intensity of spillover cases per spatial unit was explained by the climatic suitability for the two key reservoir hosts previously modelled with Maxent (Phillips et al. 2006) by Martin et al. (2016), and a subset of the Worldclim bioclimatic variables (bio1-19) (Hijmans et al. 2005). To select the explanatory bioclimatic variables less likely to negatively affect model transference to climate change conditions for each spillover system, we performed a Niche views procedure. It consists of an analysis of the correlations between pairs of explanatory variables and the location of the presence points within the bivariate clouds of data points [BIO1-18 and *P. alecto* or *P. conspicillatus* models (Owens et al. 2013)]. The variables less likely to affect model transference to climate change conditions are those where the presence points lie close to the centre or relatively far from the margins of the range of values within the bivariate cloud of data (Owens et al. 2013). This may have increased model complexity by excluding variables with more explanatory power.

### Selecting Point Process Model Structure

Parameters for the Poisson point process model and spatial autocorrelation with a log-Gaussian Cox process were sampled with a Metropolis-adjusted Langevin algorithm implemented in the R 3.2.3 ‘lgcp’ package (Taylor et al. 2013; R Core Team 2016). Model selection was performed a priori with a Poisson regression (Taylor et al. 2013). The response variable for this part of the process was the number of points per spatial area unit (e.g. pixels). We began model selection with four model structures for the Poisson regression of the *P. alecto* system; interactions between all explanatory variables; with linear, quadratic and cubic terms; and linear and quadratic terms and a series of interactions between linear, squared and cubed terms. Each model was then subject to automated step-wise variable deletion until we obtained the model with the lowest AIC. We kept the model structure with the lowest AIC. Then we tested the performance of this model structure on independent data with the partial ROC test [described in more detail below (Peterson et al. 2008)]. If the lowest AIC model performed better than random according to the test, then we used its formula in the final Poisson point process. For the *P. conspicillatus* system, we only selected one out of three models given the small number of spillover cases potentially arising from this species—all linear terms, and linear with quadratic and cubed probability of *P. conspicillatus* presence (from Martin et al. 2016).

### Finding Appropriate Priors and Covariance Function for the Bayesian Model

To find the appropriate spatial covariance function, we ran short chains of 5000 iterations of the MCMC sampler, with 500 burn-in iterations and thinning rate of 15 with the exponential and spiked exponential covariance functions. The chains were run with a range of priors and number of neighbouring cells to compute the covariance function. When the MCMC chains appeared to be well mixed, with lower values of autocorrelation and no rejected samples during the *run* phase, we ran the full MCMC chains. The final size of the full-length chains was chosen based upon the behaviour of the *h* parameter. For runs with *h* ≈ 0.3, we used 5 M iterations, but in cases where *h* decreased, the number of iterations was increased up to 20 M. Parameter priors that resulted in *h* < 0.1 at the end of the 5 K iteration chain were rejected. The burn-in phase of the full-length chains included 10% of the number of iterations and the thinning rate was set up to keep 1% of the posterior samples (Taylor et al. 2015). To have a model that represents the underlying risk, regardless of the underlying population at risk, we corrected for the effect of horse population on spillover intensity per unit area. This was done by using a horse population density model as a Poisson offset. In the absence of more data regarding horse densities than the 2007 horse census (Moloney 2011), a critical assumption in the approach is that the horse population of 2007 is still correlated and broadly representative of the horse population during the time when HeV spillover events have occurred, from 1994 to 2016 at the spatial scale of the model. Further justification for this approach is that we aimed to represent the underlying risk for spillover regardless of the density of the spillover host. This is partly because we do not have a reliable model for future horse densities. Hence the Poisson component of the model with population offset and spatial covariance results in the following Bayesian model: Poisson component:

$$ \begin{array}{*{20}c} {\mu (s) = \exp \left( {\beta Z(s) + Y(s)} \right)} \\ {R(s) = C_{A} \lambda (s)\mu (s)} \\ {X(s)\sim{\text{poisson}}(R(s))} \\ \end{array} $$

Spatial covariance function:

1 $$ \begin{array}{*{20}c} {\text{cov} \left[ {Y\left( {s_{1} } \right),Y\left( {s_{2} } \right)} \right] = \sigma^{2} r\left( {s_{2} - s_{1} ,\varphi } \right)} \\ {r(s) = \exp \left( { - s/\varphi } \right)} \\ {s = {\text{euclidean}}\left( {s_{2} ,s_{1} } \right)} \\ \end{array} $$

Parameter priors:

$$ \begin{array}{*{20}c} {\beta \sim {\text{normal}}\left( {0,10^{6} } \right)} \\ {\log \left( \varphi \right) \sim {\text{normal}}\left( {\log (500),2} \right)} \\ {\log \left( \sigma \right) \sim {\text{normal}}\left( {\log (1),0.15} \right)} \\ \end{array} $$

where β is the vector of effects of environmental covariates *Z*(*s*); *Y*(*s*) is the bivariate (*s*_1_, *s*_2_) covariance function, *C*_*A*_ is the spatial grid cell size, *λ*(*s*) is the horse population density, *X*(*s*) is the point intensity data, and *σ* and φ are the exponential covariance function *r*(*s*) parameters in Eq. (1) (Diggle et al. 2013; Taylor et al. 2013).

**Figure 2:**
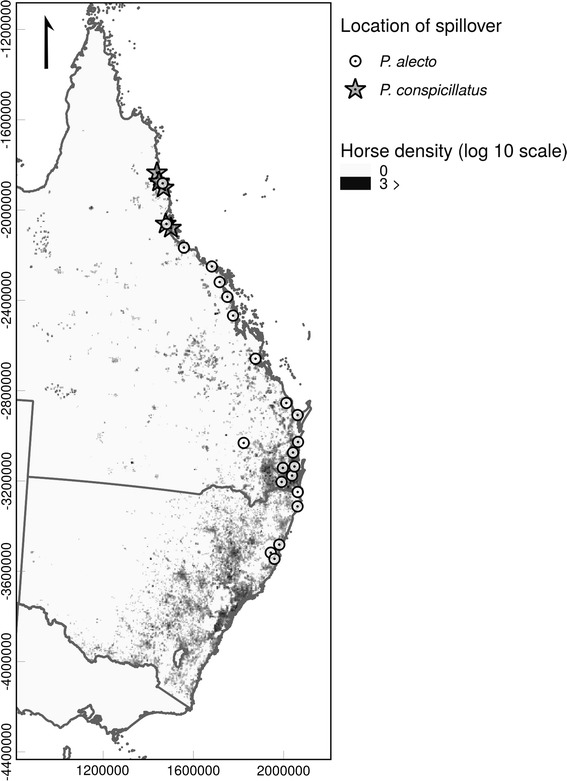
Location of spillover events overlaid on the horse density model (log_10_ scale). The symbol for spillover events represents the reservoir host species that we attributed spillover events. Spillover localities were thinned to improve visualisation.

The horse population density model was built with the horse population census of 2007 (Moloney 2011). This horse density model was created by introducing uniformly distributed noise to the geographical coordinates of the horse properties, equivalent to 50% of the cell width of the environmental data. The number of horses per grid cell after noising the coordinates was rasterised, and the process was repeated 100 times. When iterations were completed, the 100 raster layers were averaged to create the final horse density model (Fig. 2). This method allowed us to account for the effect of properties spanning more than one grid cell but whose centroid lied within a single pixel.

### Transferring the Model to Climate Change Scenarios

Once we obtained the converged MCMC chains, we used the posterior estimates of the environmental covariates to project the point intensity of spillover per unit area in geographic and environmental space. For the final spillover risk model, we calculated the probability that the predicted intensity exceeds the lower 20^th^ percentile of the median intensity estimated for the locations with spillover events. This threshold was chosen because the database contains location uncertainty in nearly 20% of the spillover locations. Before we simulated future scenarios of spillover, we compared the data used for model fitting with the data from climate change predictions with an extrapolation analysis [ExDet (Mesgaran et al. 2014)]. Briefly, extrapolation analyses consist of finding extended variable ranges (type 1 extrapolation) and different correlation structures (type 2 extrapolation) that might affect the behaviour of the statistical model. These analyses are performed with the raster data used for model transference and result in highlighting geographical areas where the model might misbehave. We used the extrapolation analysis results to remove all areas where models faced extrapolation due to novel climatic conditions. We used the 16 climate change scenarios under two different greenhouse gas representative concentration pathways [RCP 45 and 85 (Hijmans et al. 2005)]. This approach has been suggested to represent degrees of confidence in the potential outcomes of climate change given the variability between global circulation models, RCPs and downscaling methods (Beaumont et al. 2008). Concentration pathways are a series of alternative trajectories that greenhouse gas concentration might follow depending on a series of ecological, technological, socio-economic, political and demographic factors that could result in different degrees of climate change. The number that accompanies the acronym RCP indicates the severity of greenhouse gas concentration (van Vuuren et al. 2011).

To generate the climate change scenarios consensus maps, we began by setting a threshold of 0.2 for all exceeding probabilities to coincide with the threshold that was used to calculate the probabilities and test the models (see below). The areas predicted absent with this threshold under current climatic conditions were set to -1 (presence = 1, absence = − 1). Then, each of the thresholded climate change projections had 1 added, so that areas predicted absent = 1, and areas predicted present = 2. After adding up all the thresholded predictions, we subtracted the probability that there was model extrapolation to force all extrapolated areas back to 1 (or between 1 and 2 if not all climate change scenarios resulted in extrapolation for that area). Finally, we multiplied by the values for the current risk scenario based on current climatic conditions. For this, areas predicted absent using the 0.2 threshold were set to -1 and presence to 1 (absence = − 1, presence = 1)). This multiplication resulted in all areas that were predicted present in future climatic conditions but were absent in today’s conditions becoming negative. All areas that were predicted present both now and into the future got positive values between 1 and 2; and the areas that became unsuitable after climate change received values close to 1 (presence today (1) × absence future (1) = 1). Future absence in the areas represented by these calculations was caused by areas remaining in the same absent state as currently, areas becoming unsuitable, or by being unable to predict anything due to extrapolation. To help readers identify areas where predictions are dubious due to extrapolation we have provided a map of extrapolation conditions.

Models’ predictive performance was measured with the partial ROC (receiver operator characteristic) with a 20% omission rate (Peterson et al. 2008) for the *P. alecto* system, and with a jackknife test (Pearson et al. 2006) for the *P. conspicillatus* system. The partial ROC test is a modification of the traditional ROC area under curve (AUC) that measures a model’s ability to discriminate zeroes from ones. An AUC score of 1 means the model is capable of perfect discrimination (no false positives or negatives). The partial ROC, however, is based on the spatial performance of the model projection by contrasting the percent of area predicted that is used to generate a random predictor with the proportion of presences predicted. The resulting score is the quotient of the model’s AUC and the random predictor’s AUC. Consequently, the maximum partial ROC score is two (Peterson et al. 2008). To run this test with independent data, we partitioned the *P. alecto* system data in four sets and cross-validated their predictions with models fit in one chain of 5 M iterations, burn-in of 500 K and thinning of 4.5 K. We allowed the partial ROC test a 20% omission of the test points to represent the 20% uncertainty in spillover location according to the Biosecurity Queensland dataset. While desirable, a higher number of partitions was not possible due to the high computational intensity of these analyses. For the *P. conspicillatus* system, we fitted 8 models, each one omitting one of the spillover presence points. Then we calculated the minimum thresholds and their corresponding per cent of area predicted for the jackknife test (Pearson et al. 2006).

## Results

### Predicted Distributional Patterns

**Figure 3:**
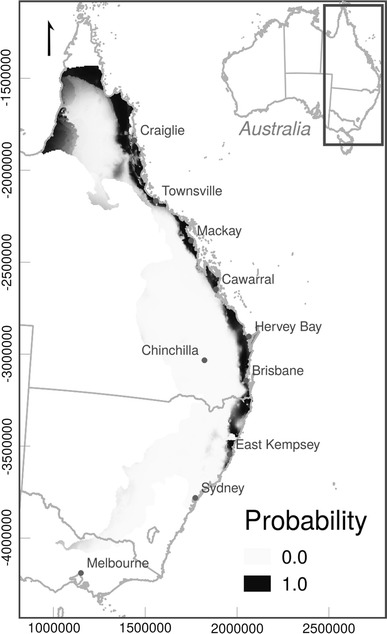
Present probability of exceeding the intensity threshold of Hendra virus spillover in the *P. alecto* system.

**Figure 4:**
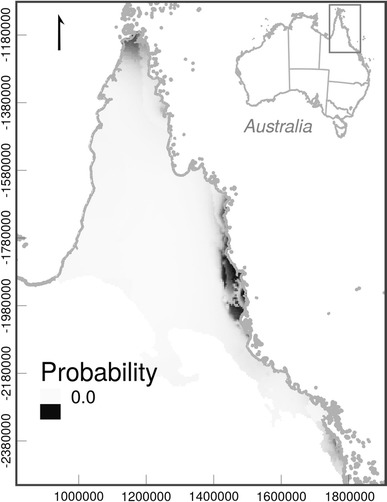
Present probability of exceeding the intensity threshold of Hendra virus spillover in the *P. conspicillatus* system.

The predicted spatial patterns of HeV spillover risk under present climatic conditions for both reservoir host systems, *P. alecto* and *P. conspicillatus*, are consistent with the distribution of spillover events since 1994. Current risk, as explained by *P. alecto*, comprises most of the east coast of Australia, from northern Queensland to central New South Wales, and overlaps with parts of the distribution of *P. conspicillatus*. Additional areas spanning farther north than the northernmost known spillover event were also predicted to be at risk (Fig. 3). Risk driven by *P. conspicillatus* resulted in novel areas predicted to be at risk of spillover, located in the northernmost location within Australia (Fig. 4).

**Figure 5:**
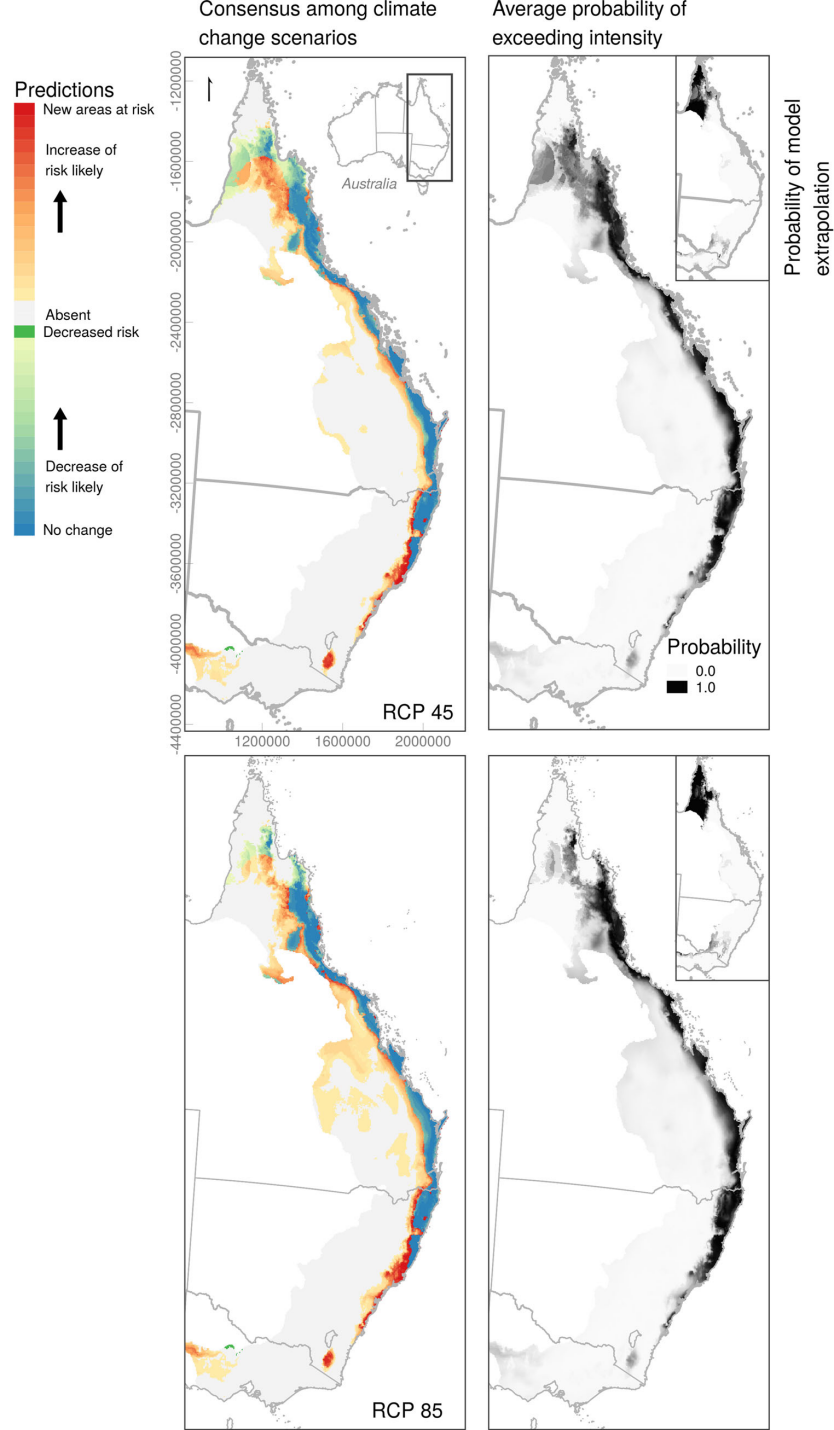
Predicted distribution of spillover for 2050 in two greenhouse gas representative concentration pathways (RCP) for the *P. alecto* system. Top RCP 45, and bottom RCP 85. Left panels show areas of expansion and contraction and the level of agreement between climate change scenarios. Right panels, the average predicted probability of exceeding spillover intensity among climate change scenarios (main panels). Top right corners show the probability of model extrapolation.

**Figure 6:**
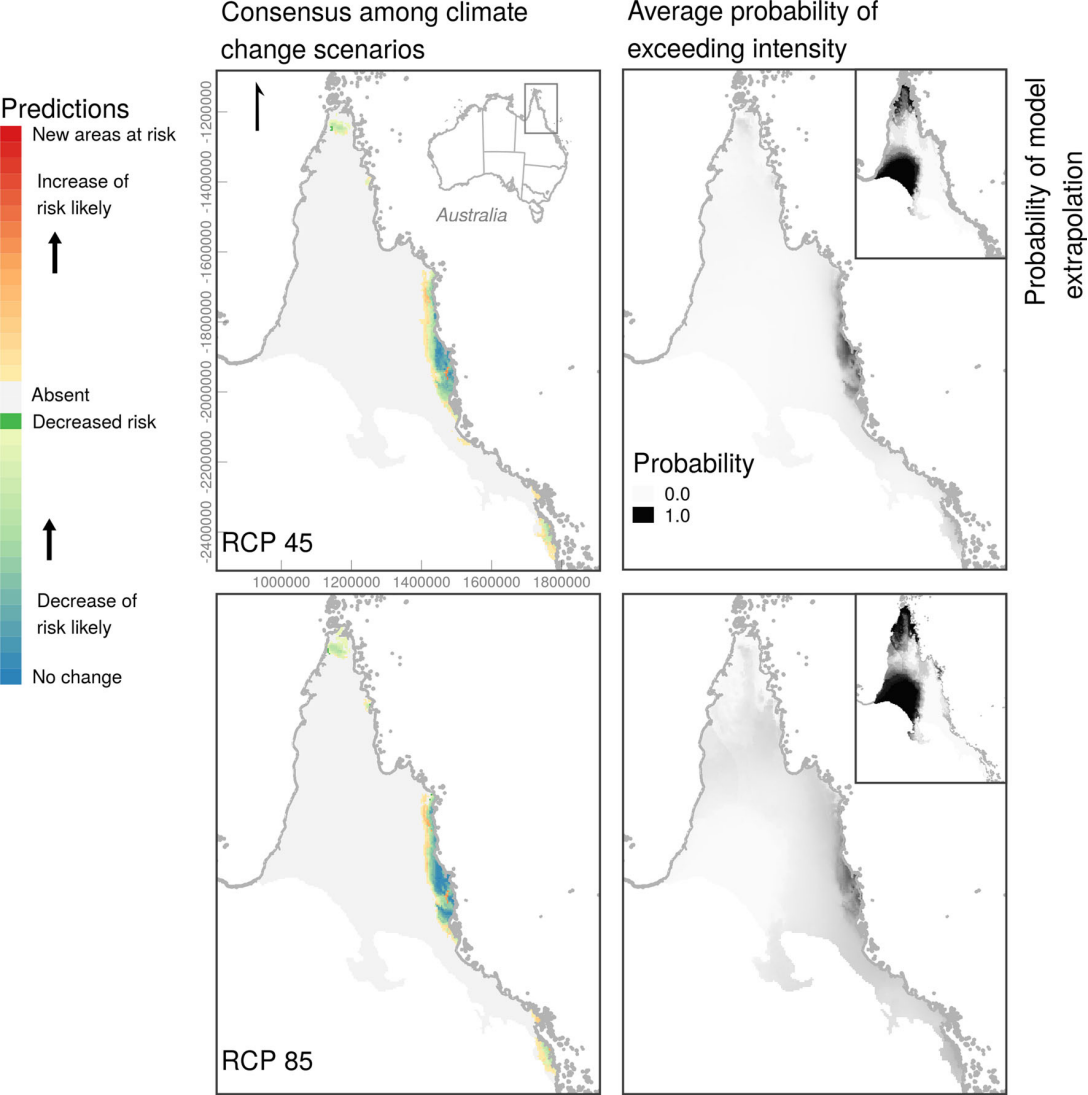
Predicted distribution of spillover for 2050 in two greenhouse gas representative concentration pathways (RCP 45 and 85) for the *P. conspicillatus* system. Left panels show the areas of contraction and expansion and the degree of agreement between climate change scenarios. Right panels show the averaged probability of exceeding the spillover intensity threshold. Top right corner of right panels shows the pixel-wise probability of extrapolation.

In the *P. alecto* HeV spillover system, when we projected the models to future climate change scenarios in 2050, all 16 scenarios agree that there could be an expansion of risk towards the south and slightly farther inland (red areas in left panels of Fig. 5). At current horse population levels (estimated during the census 2007–2011), the areas predicted to experience significantly greater risk under climate change scenario RCP45 (greenhouse gas representative concentration pathway 45) contain up to 112 914 horses, and 164 391 horses for RCP85. These numbers represent a 175–260% increase in the horse population at risk. The majority of horses at increased risk occur over 390–425 km of coastline south of the southernmost known spillover event (Kempsey, Figs. 2, 3). Expansion of risk under both RCPs reaches the Hunter Valley (west of Sydney) which has a high density of horses. The climate change scenario agreement in this area is very high. Despite the increased geographical extent of spillover risk model projections predicted average lower probability of exceeding the specified intensity threshold for spillover to occur (20th quantile of median intensity at spillover locations) compared with current conditions (Figs. 5, 6).

In northern areas, there is consensus that there could be greater risk levels in novel areas in the *P. alecto* spillover system. However, most of these areas could face novel climatic conditions that would result in model extrapolation, which increases uncertainty (top right corner of right panels in Fig. 5). We could not identify any areas that would become completely unsuitable for spillover (marked with green). The only areas with 100% agreement to become completely free of risk are most likely over predictions because they lie very far from the known distribution of spillover and *P. alecto*.

The major difference between the greenhouse gas representative concentration pathways is a greater inland expansion of areas at risk in RCP 85 compared with RCP 45 based on the *P. alecto* HeV spillover system. This means that in the more severe climate change scenario risk could increase farther inland.

With respect to *P. conspicillatus*, models predicted both expansion and contraction but with low agreement between climate change scenarios (left panels Fig. 6). The northernmost end of areas at risk were predicted to shrink in both concentration pathways, although these areas were affected by extrapolation. Other small areas were predicted to become unsuitable in RCP 85 that were not affected by extrapolation. Both scenarios, RCP 85 and 45, predict lower probabilities of exceeding the intensity threshold compared with current climate scenarios (right panels in Fig. 6). The areas that experienced no change for *P. conspicillatus* were small, and compared with *P. alecto* they experienced either no change or expansion (darkest blue Figs. 5, 6). This indicates that *P. conspicillatus* habitat is likely to shrink and become more suitable for *P. alecto,* which raises concerns over *P. conspicillatus* conservation measures that might also affect HeV epidemiology.

### Model Fit and Performance

The model selection procedure resulted in retention of the variables and interactions in Tables 1 (*P. alecto*) and 2 (*P. conspicillatus*). For the *P. alecto* system, we could successfully implement the best model according to the AIC of the Poisson regression. However, for the *P. conspicillatus* system all attempts to include linear and quadratic terms or their interactions resulted in either singularity of the covariance matrix or in very long MCMC chains. As a result, we sought to keep a balance between AIC and the convergence properties of the MCMC chain which resulted in a simple model of linear terms of the climatic components and a cubic term of *P. conspicillatus* climatic suitability. Estimates of the regression coefficients (β) and spatial component (φ, σ) are listed in Tables 1 and 2.

**Table 1:** Parameter Estimates of the *P. alecto* System Model.

Parameter	Median	Credible intervals	
		2.5%	97.5%
log(σ)	3.817497 × 10^−2^	− 1.568415	7.081970 × 10^−1^
log(φ)	1.588467	2.679587 × 10^−1^	3.093003
β_Intercept_	3.922981	− 3.376057 × 10^1^	3.852942 × 10^1^
β_bio5_	− 9.316293 × 10^−2^	− 1.890911 × 10^−1^	1.705975 × 10^−2^
β_bio9_	− 8.620517 × 10^−3^	− 5.012380 × 10^−2^	3.087590 × 10^−2^
β_bio12_	− 1.710871 × 10^−2^	− 3.934777 × 10^−2^	3.480018 × 10^−3^
β_bio15_	1.840011 × 10^−1^	9.811339 × 10^−2^	2.795649 × 10^−1^
β_Maxent.p.alecto_	− 1.438959 × 10^2^	− 2.504255 × 10^2^	− 3.564127 × 10^1^
β_I(Maxent.p.alecto^2)_	5.913680 × 10^1^	− 6.198367	1.252079 × 10^2^
β_bio5:Maxent.p.alecto_	2.524097 × 10^−1^	4.738640 × 10^−2^	4.704084 × 10^−1^
β_bio12:Maxent.p.alecto_	9.334252 × 10^−2^	2.171861 × 10^−2^	1.726844 × 10^−1^
β_bio12:bio15_	− 1.433364 × 10^−4^	− 7.637361 × 10^−5^	− 2.197990 × 10^−4^
β_bio12:I(Maxent.p.alecto^2)_	− 7.449967 × 10^−2^	− 1.408666 × 10^−1^	− 1.353857 × 10^−2^

β’s represent the regression coefficients in exponential scale. Parameters σ and φ are the mean and variance of the spiked exponential covariance function.

**Table 2:** Parameter estimates of the *P. conspicillatus* system.

Parameter	Median	Credible intervals	
		2.5%	97.5%
log(σ)	0.2876164	2.29218438	1.72870463
log(φ)	1.5956162	0.17490535	2.93283772
β_bio2_	− 0.3454483	− 0.82917878	− 0.06260373
β_bio5_	0.1196481	− 0.09607769	0.48394434
β_bio9_	− 0.1569954	− 0.48183344	0.04463800
β_I(p.consp^3)_	10.3125608	2.46454087	28.37984947

Both models converged with 10 M iterations, burn in of 1 M and thinning rate of 9 K. Both models performed better than random based on their respective performance metrics. The *P. alecto* HeV spillover system had an area under the curve ratio of 1.47 that was significantly different from 1 (*P *= 0.04, 1 represents the random prediction threshold) with an omission rate of 20% consistent with the threshold to calculate the exceeding probabilities. The *P. conspicillatus* spillover system that was tested with the jackknife test also performed better than random with a prediction rate of 0.75 (*P* = 2 × 10^−6^). Given that we were looking for a 20% omission threshold and a small number of presence points in the *P. conspicillatus* data set, the prediction rate of 0.75 is acceptable, because in a binomial process with the same sample size it is not significantly different from 0.8 (*P *= 0.99).

The models that were tested during the model structure selection procedure performed better than random. The AUC ratios of partitions were 1.84 (SD = 0.069, *P* = 0) and 1.85 (SD = 0.053, *P* = 0) with the same 20% omission rate.

### Extrapolation Analysis

All future climate scenarios result in novel conditions for the models especially in northern areas. Extrapolation affected mostly the projections of the *P. alecto* HeV spillover system and occurred in the novel areas in northern Queensland (top right corner of the right side panels Fig. 5). All the climatic variables used in the model caused type 1 novelty in these areas. Type 1 novelty is an increased range of values than used in model training. Additional areas of extrapolation occurred in southern locations along the coast. Because extrapolation in these areas was caused by the model of *P. alecto* distribution, which can only have values 0–1, extrapolation artefacts are unlikely. We did not perform an extrapolation analysis for the *P. alecto* distribution models, but only compared the predictions of Maxent models fit with and without clamping and extrapolation and did not notice any differences.

Extrapolation affecting the *P. conspicillatus* system was mostly present in areas accessible to the species, but that are outside the areas estimated as suitable for *P. conspicillatus*. Therefore, extrapolation is unlikely to affect predictions save for northernmost locations in the study area (Fig. 6). However, some climate change scenarios predict the occurrence of extrapolation type 1 and 2 likely caused by *P. conspicillatus*. Probability of extrapolation for this system is shown in the top right corner of the right side panels in Fig. 5.

## Discussion

Under climate change, suitability for HeV spillover could expand southwards. In addition to a southward expansion, some scenarios predicted inland expansion in the *P. alecto* HeV spillover system. However, while the total area at risk of spillover was predicted to increase, the average probability of spillover in these areas could slightly decrease, especially in the *P. conspicillatus* system. There was high uncertainty of future risk in areas north of the current distribution of spillover. In areas currently inhabited by *P. conspicillatus, P. alecto* was predicted to remain stable or expand. In areas where both *P. alecto* and *P. conspicillatus* were predicted to co-occur, average probability of exceeding the intensity threshold was predicted to decrease with respect to both species. *P. alecto*’s expansion indicates that additional mitigation efforts should be allocated where risk has been predicted to increase (marked as red in the consensus maps in Figs. 5, 6). In addition, the expansion of *P. alecto* into *P. conspicillatus* territory suggests that *P. alecto* may replace or become the more predominant HeV host in those areas.

The current forecasted area at risk of HeV spillover to horses is wider than the area that contains the detected HeV spillover events. Based on both *P. alecto* and *P. conspicillatus,* areas farther north than previously recognised were predicted to be at risk. Absence of spillover detection in these areas is probably due to the very low density of horses (Fig. 2) and relative lack of disease surveillance. While the effect of horse density on risk of spillover seems negligible (McFarlane et al. 2011) or negative depending on the spatial scale (Martin et al. 2018), the presence of horses is conditional for spillover (Plowright et al. 2015).

Previous niche modelling studies of *Henipavirus* hosts predicted broad areas at risk in Australia (Daszak et al. 2013). Our results differ from these predictions because we used the actual spillover events to fit the model and because we narrowed down the number of reservoir hosts from four to two. Before 2014 it was unclear which bat species were more relevant for HeV epidemiology. Recent findings have provided epidemiological (Smith et al. 2014; Edson et al. 2015), and ecological (Martin et al. 2016) support for *P. alecto* and *P. conspicillatus* as the main HeV reservoir hosts. Hence, we have predicted smaller areas at risk.

Poisson point processes have infrequently been used to model the spatial pattern of spillover of bat-borne viruses. Walsh (2015) modelled the spatial pattern of Nipah virus spillover to humans as a point process model in response to human footprint, the presence of bat reservoirs and environmental factors (vegetation). One key difference with our study is that we focused on modelling HeV spillover driven by environmental factors in order to be able to project the models into climate change scenarios. This enabled us to explore the potential consequences of climate change for HeV spillover. With that in mind we used reservoir host density (statistically equivalent to the human footprint variable in Walsh (2015)) as an offset term to specifically model the isolated effect of climate. We decided to take this approach because of the lack of data to predict future horse density and distribution, which precludes its inclusion as a HeV spillover predictor.

Previous studies of the zoonotic niche of bat-borne viruses including Marburg and Ebola viruses (Peterson et al. 2004, 2006; Pigott et al. 2014, 2015) and Nipah virus (Peterson 2013) have used machine learning methods. Interpretation of these models and the risk management implications of the predictions were thus limited to visual analysis of the geographic patterns and associated climatic factors. The transparent nature and control over model selection that Poisson point processes confer result in better understanding of the likely biological meaning of statistical associations (Renner et al. 2015; Taylor et al. 2015). However, definitive interpretation is dependent on understanding of the underlying biological mechanisms (Walsh 2015).

Although the final models are complex due to a lack of understanding of the interaction of flying foxes and horses, the statistical associations in the models of the *P. alecto* system are similar with those found in Martin et al. (2018). First, most of the variables’ interactions that were kept in the model represent rainfall (*bio12*) and its seasonality (*bio15*). These two interact with *Maxent.p.alecto* indicating that interactions between rainfall, its variability, and the probable presence of this bat species are important for spillover. Such effects could be due to the climatic differences between areas used for foraging and establishing a roost (Tidemann et al. 1999; Vardon et al. 2001). In fact, high suitability for *P. alecto* is not enough to explain spillover because *Maxent.p.alecto* alone had a negative effect which is reversed when it interacts with rainfall levels (Table 1). We can infer from these associations that rainfall levels and their variability with respect to seasonal extremes could be broad-scale correlates of HeV spillover risk (Páez et al. 2017; Martin et al. 2018).

In the *P. conspicillatus* system, the small number of spillover events, nine, limits the number of variables and their interactions that could be included in the model. From the final model structure only *Maxent.p.conspicillatus* and *bio2* (mean diurnal temperature range) had significant effects (Table 2). Given the cubic exponent affecting the positive effect of *Maxent.p.conspicillatus*, we infer that transmission from this species to horses occurs in areas where climatic suitability is very high for *P. conspicillatus*.

There was complete agreement among climate change scenarios that there could be a southward increase in suitability for spillover caused by the response of *P. alecto* to climate change. However, the already observed southward expansion of *P. alecto* is faster than predicted by changing climate (Roberts et al. 2012), suggesting that other non-climatic factors like urbanisation (Plowright et al. 2011; Tait et al. 2014) are also affecting the presence and density of the bat species. To date the southernmost recorded spillover events lie within the limits of the current potential distribution of spillover (blue areas in left panels of Fig. 5). This shows that even when *P. alecto* is capable of occupying areas beyond its optimal climatic niche, spillover and spillover risk occur within the areas with the highest climatic suitability in most cases, most likely due to higher potential densities of *P. alecto* [climatic suitability is correlated with bat density and spillover risk (Smith et al. 2014; Martin et al. 2016)]. Hence, as climatic suitability for *P. alecto* continues to increase southwards, potential for higher population densities could increase southwards as well as HeV spillover risk.

The cause of predicted expansions under climate change with high agreement might be related to the higher temperatures expected at higher altitude and lower latitudes (Lafferty 2009), particularly in Australia (Williams et al. 2003). This is consistent with the predictions of tropical diseases expanding or shifting into subtropical areas (Lafferty 2009). The lower agreement on the inland expansion indicates that the effect of altitude is less clear among climate change scenarios. In fact, some of the scenarios indicate that there could be a contraction towards the coast. Consequently, to adequately assess if there will be expansion or contraction to and from the coast, flying fox monitoring programs are required.

In model projections, we identified overpredictions (Figs. 3, 5). These could be due to the inclusion of areas that are not usually available to *P. alecto* (Soberón and Peterson 2005). Accessible areas are usually defined by physical barriers, however, in the absence of such evident barriers for pteropodid bats in Australia we assumed that climate could act as a barrier through its effects on bat physiology. While the assumption could be valid, the choice of climatic regions clearly did not eliminate inaccessible areas that could be suitable, at least according to some climatic factors. Alternatively, the most relevant climatic factors that restrict the distribution of bats and HeV spillover might have been discarded in the search for variables that were less likely to impact the model’s transference to climate change scenarios (Owens et al. 2013).

The ultimate implications of the southward and probable inland expansion are a greater number of horses at spillover risk. Depending on the representative concentration pathway (RCP), and based on the 2007 horse census, there could be at least 112–165,000 more horses at risk (175–260% increase). Because there is considerable uncertainty around the potential outcomes of climate change on disease occurrence in new areas more research is needed, first to verify predictions and then to better manage the consequences (Braks et al. 2013). Furthermore, the ultimate spillover risk scenario by 2050 will also depend on horse densities and socio-economic processes, and how these processes interact with climate change. Therefore, one potential area of ecological and epidemiological research is the role of novel ecological interactions between flying foxes and other organisms such as food sources that could experience distributional shifts and impacts as result of human activities. We need to understand if these novel interactions and processes affect the dynamics of bat populations, HeV, and spillover risk (Williams et al. 2003; Sala et al. 2009). Consequently, we emphasise the need to undertake regular risk assessments to quantify HeV exposure in horse populations and to consider the potential consequences of a larger horse population at risk.

In light of the potentially larger horse population at risk, it is clear that direct intervention of the HeV spillover system is necessary to mitigate risk in response to climate change such as extending vaccine coverage regardless of the uncertainties involved (Beaumont et al. 2008). However, a more holistic approach would include reduction of greenhouse gas emissions. Such a management strategy would positively impact all levels of organisation of the HeV spillover system studied here and prevent other predicted consequences of climate change. For instance, the Australian tropics are predicted to experience large biodiversity losses (Williams et al. 2003) and grasslands in southern Australia could experience increased variability in productivity which could affect the cattle and potentially the horse industry (Cullen et al. 2009).

Our models predicted that spillover frequency could decrease in response to climate change with respect to *P. conspicillatus*. However, the *P. alecto* HeV spillover system was predicted to remain or increase within the current areas suitable for *P. conspicillatus*. Therefore, these areas of tropical north Queensland could experience a replacement of reservoir host species that may result in different epidemiological processes that would benefit from different mitigation strategies. We recommend, therefore, that an area of research be the development of specific management strategies for the different flying fox species relevant to Hendra virus spillover. These management strategies would anticipate and better manage flying fox species’ replacements and changes in the epidemiology of HeV spillover.

The predicted shrinking of the distribution of *P. conspicillatus* could also affect the dynamics of many ecosystem processes because flying foxes are important pollinators and seed dispersers. The absence of such ecosystem services could result in further biodiversity loss. Such loss of ecosystem services occurs even before bats become extinct (McConkey and Drake 2006). Therefore serious additional conservation issues may arise as a result of *P. conspicillatus* decline that could affect HeV epidemiology.

Predicting HeV spillover with the methods we used carries considerable uncertainty. Sources of uncertainty may be related to: (1) the type of presence only data used limits the number of analytical methods that can be used and hampers the identification of limiting factors; (2) the effects of climate on HeV spillover act at several different levels of ecological organisation and are not well understood, for instance temperature, humidity and ground vegetation might also limit the available pathways for HeV transmission to horses (Martin et al. 2017), and temperature can regulate the flowering status of native plants (Hudson et al. 2010), the main source of food for flying foxes; (3) flying fox species distributions do not depend entirely on climate (Tidemann et al. 1999; Vardon et al. 2001), but are greatly affected by native plant phenology (Giles et al. 2016), and have an apparently innate preference for fragmented and urbanised landscapes (Tait et al. 2014); (4) the predicted distributions of flying foxes in response to climate change do not account for other organisms’ shifting distributions that affect bats’ distributions. Other organisms shifting distributions might give rise to novel and unpredictable interactions and effects on bats’ distributions (Eby et al. 1999; Eby and Law 2008; Giles et al. 2016); and (5) climate change could also affect horse behaviour and susceptibility to diseases. For instance, horses have limited thermal tolerance. Exceeding their comfort levels can alter their behaviour (Castanheira et al. 2010) and increase the frequency of interaction with tree shaded areas (Jørgensen and Bøe 2007), which is where HeV is usually excreted (Field et al. 2011). All of these issues warrant further research to increase understanding of HeV epidemiology and bat virus spillover in general.

The strength of our approach lies in its generality. Possible improvements to our models to make them more specific might involve: (1) including a model of bat distribution that better accounts for the effect of urbanisation (Tait et al. 2014); (2) including other biological interactions that are crucial for bat species (Giles et al. 2016) that can be transferred to climate change scenarios; and (3) establishing direct links between climatic factors and the levels of HeV infection in bats. Such an analysis would likely result in smaller areas and populations predicted to be at risk.

Spillover of diseases from wild to domestic animals and humans comprises several levels of ecological organisation. The first level includes the distribution of the reservoir host, and then the distribution of the causal agent within the reservoir host (Plowright et al. 2015, 2017). By including the additional layer of spillover host distribution as an offset during model fit, we have modelled the direct effect of climate (Taylor et al. 2015) on the biological processes that affect the reservoir and the causal agent and result in HeV spillover. Therefore, the models represent the underlying risk to any spillover hosts present in the areas predicted to be at risk due to the presence of the reservoir host and the effects of climate on the HeV spillover system. The 20% omission threshold indicates that within these areas at least 80% of spillover cases could occur. The precise location and timing of spillover cases will depend on processes that occur at finer scales like the fraction of susceptible horses (e.g. unvaccinated) that are effectively exposed (Plowright et al. 2015; Martin et al. 2015, 2017). Consequently the models should only be used to improve understanding of spillover risk, identify areas to allocate resources for mitigation and inform research activities.

## Conclusions

Our results suggest that spillover events could increase farther south, and inland with climate change. The current potential distribution of HeV spillover spans farther north, but the absence of reported events might be due to very low horse density and less disease surveillance. Spillover events could potentially increase farther south, and inland with climate change. These potential expansions and additional areas of risk should be assessed in the first instance by monitoring flying fox populations. In northern Queensland, the probable replacement of *P. conspicillatus* by *P. alecto* suggests that mitigation strategies of HeV spillover risk may have to be adapted to cope with this interaction and its uncertain effects.

## Electronic supplementary material

Below is the link to the electronic supplementary material.

Supplementary material 1 (PDF 368 kb)
