# Supplementary material for: Climate Change Could Increase the Geographic Extent of Hendra Virus Spillover Risk
Source: Ecohealth. 2018 Mar 19;15(3):509–25. doi: 10.1007/s10393-018-1322-9 (PMC6245089; doi:10.1007/s10393-018-1322-9)
Supplement: Supplementary file 1 — Supplementary material 1 (PDF 368 kb) [file 10393_2018_1322_MOESM1_ESM.pdf]

Supplementary materials

Climate change could increase the extent of areas at risk of Hendra virus spillover

Diagnostic tests of model convergence

P. alecto spillover system model

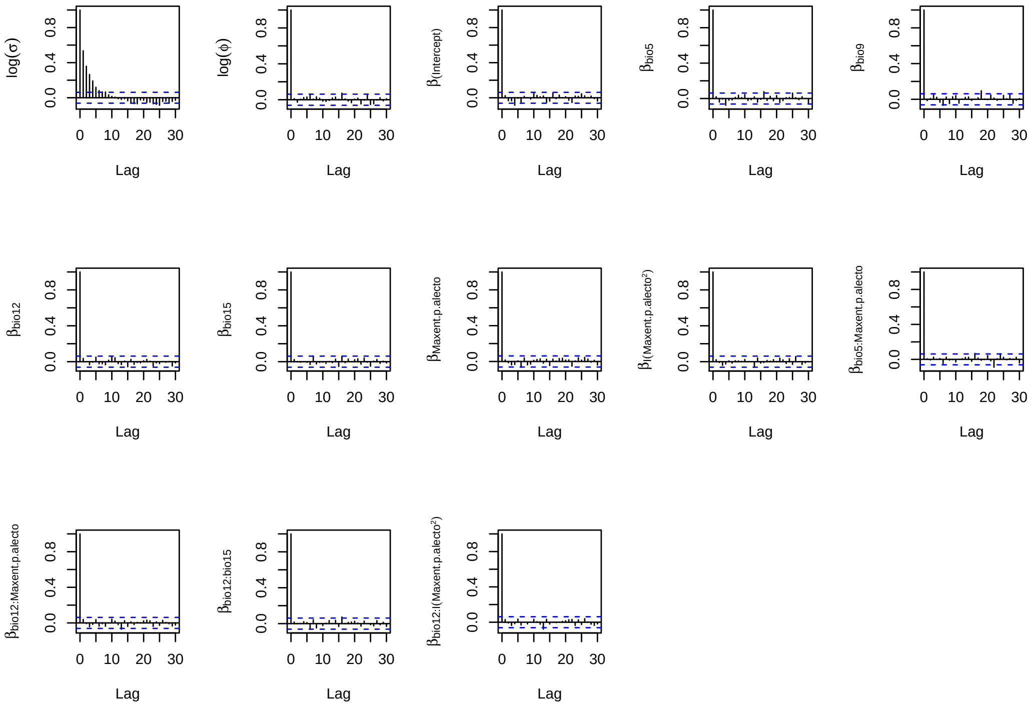

Figure S1. Parameter autocorrelation in the *P. alecto* model.

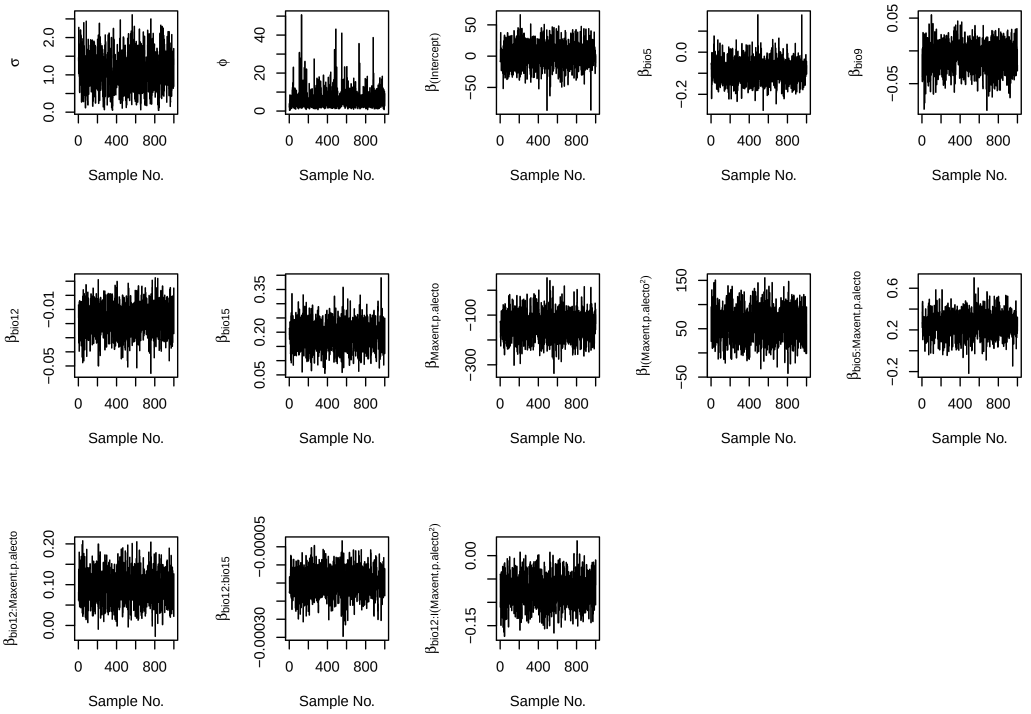

Figure S2. Trace plots of the MCMC chains of the *P. alecto* model.

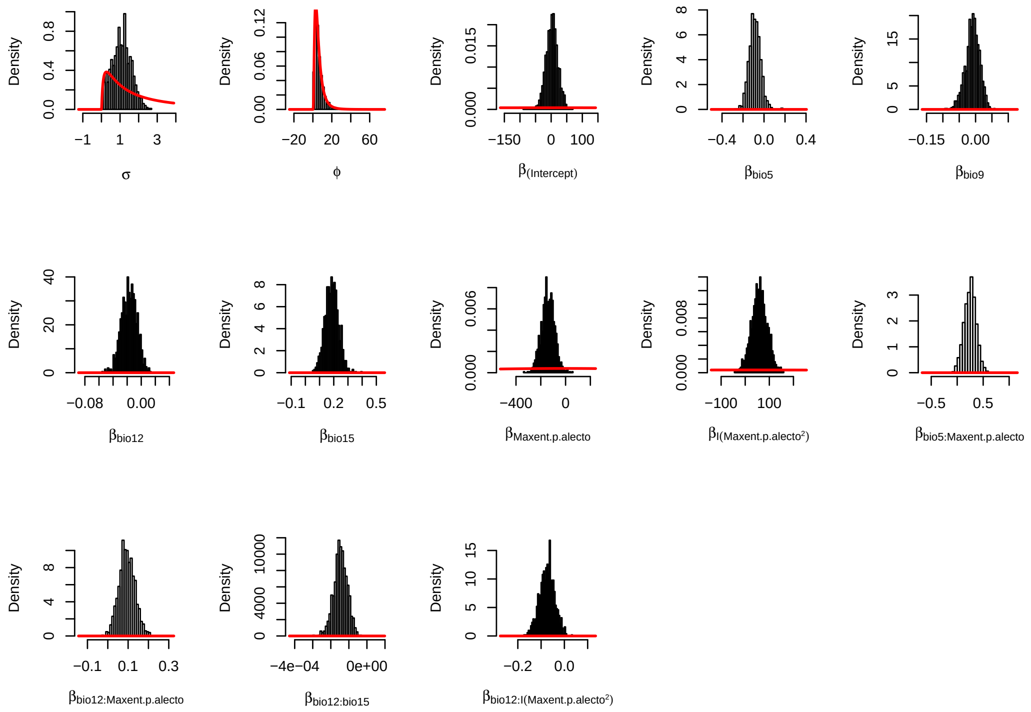

Figure S3. Priors (red density curves) and posterior estimates of the *P. alecto* model.

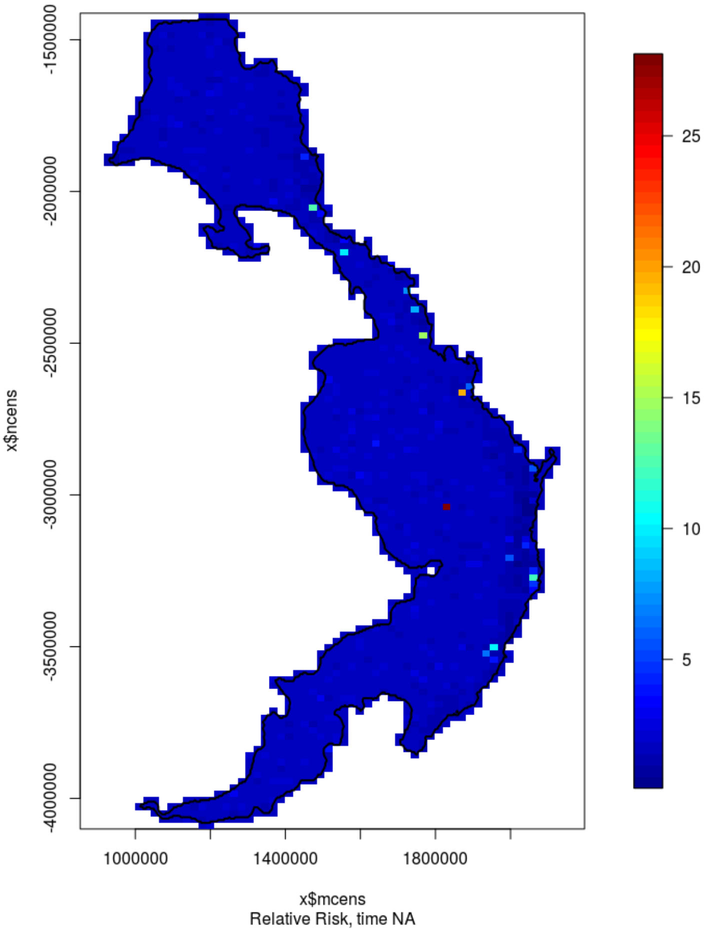

Figure S4. Spatial autocorrelation estimates (random effects) in the *P. alecto* system.

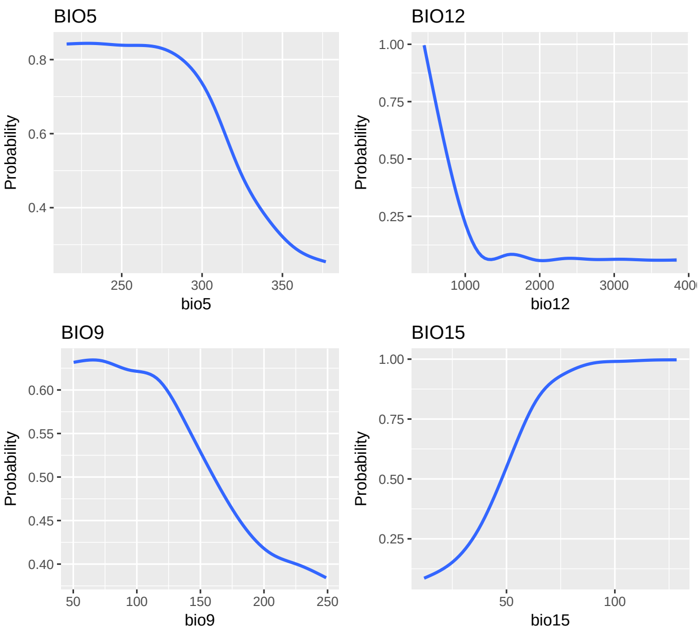

Figure S5. Individual partial responses (exceeding probabilities) in the *P. alecto* model.

P. conspicillatus spillover system model

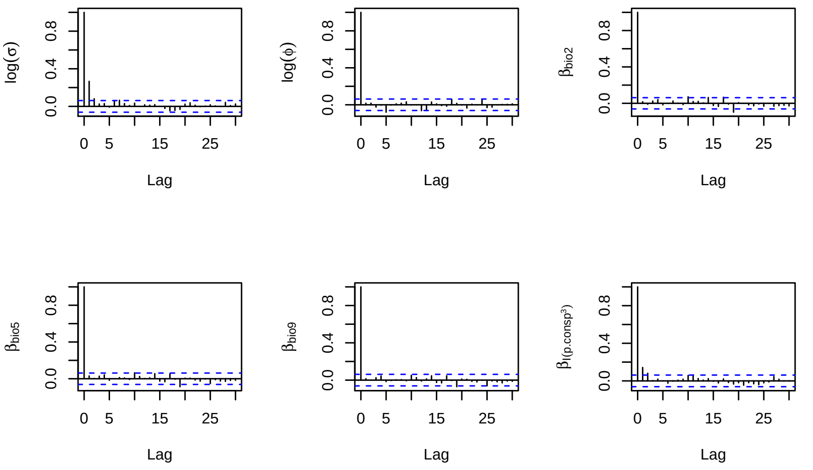

Figure S6. Parameter autocorrelation in the *P. conspicillatus* model.

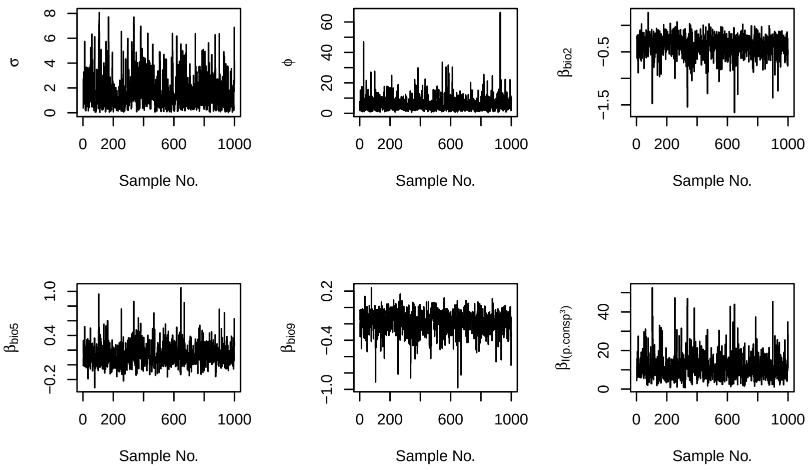

Figure S7. Trace plots of the MCMC chains of the *P. conspicillatus* model.

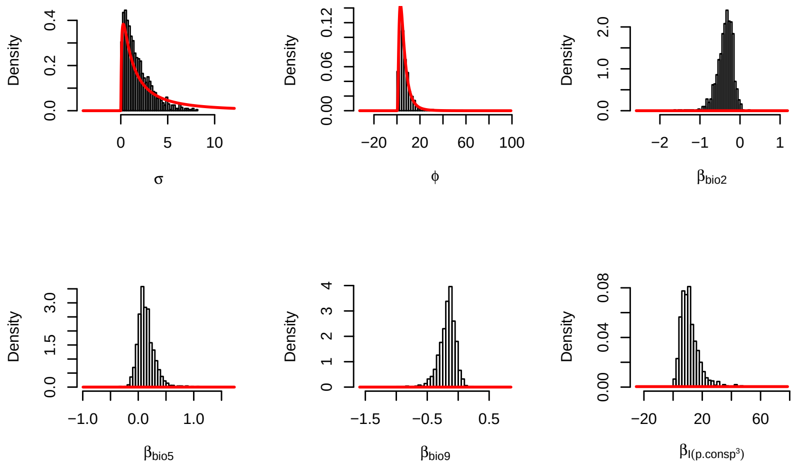

Figure S8. Priors (red density curves) and posterior estimates of the *P. conspicillatus* model.

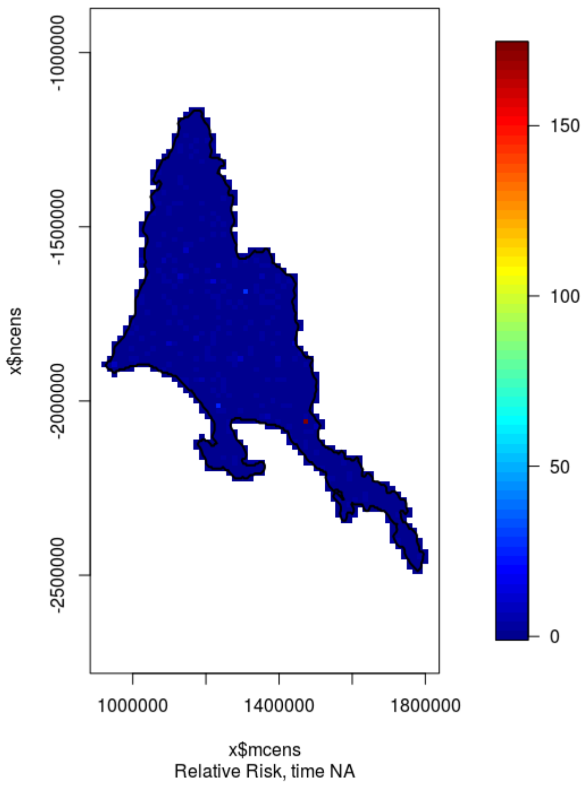

Figure S9. Risk function fitted for the *P. conspicillatus* model.

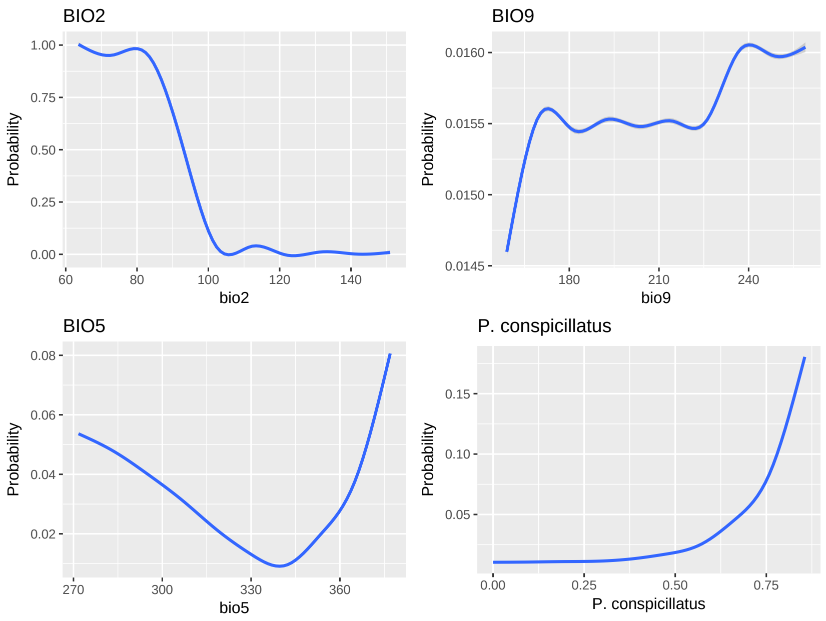

Figure S10. Individual partial responses (exceeding probabilities) in the *P. conspicillatus* model.
